# International Survey of Physicians’ Perspectives on Percutaneous Endoscopic Gastrostomy Tube Feeding in Patients with Dementia and Review of Literature

**DOI:** 10.7759/cureus.4578

**Published:** 2019-04-30

**Authors:** Naveen Mohandas, Raghu Kumar, Venkatakrishnan Leelakrishnan, Sudeep Sharma, Krishna Aparanji

**Affiliations:** 1 Gastroenterology, Mid Cheshire Hospitals National Health Service Foundation Trust, Crewe, GBR; 2 Gastroenterology, Flinders Medical Centre, Adelaide, AUS; 3 Gastroenterology, PSG Institute of Medical Sciences and Research, Coimbatore, IND; 4 Miscellaneous, University of Illinois, Springfield, USA; 5 Critical Care Medicine, Springfield Clinic, Springfield, USA

**Keywords:** peg tube, advanced dementia, physician's perspectives, complications associated with peg, percutaneous endoscopic gastrostomy, enteral access, feeding tube, nutrition, aspiration following peg, quality of life with peg

## Abstract

Percutaneous endoscopic gastrostomy (PEG) tube often remains to be used as a primary modality for feeding in patients with advanced dementia, perhaps due to misconceptions regarding the outcomes. Physicians' perceptions regarding the PEG tubes could be a significant contributing factor globally. A multidisciplinary approach involving the ethics committee can help address the issue. Our survey is focused on gauging physicians' perceptions regarding PEG tube utilization and its global impact on outcomes in dementia.

## Introduction

Approximately 50 million people are estimated to live with dementia globally today, and this number is estimated to increase to 152 million in 2050 [1]. Patients with advanced dementia often lose their ability to eat due to many reasons. They can develop oro-pharyngeal dysphagia presenting as difficulty in swallowing, loss of appetite and/or apraxia or difficulty in coordinating movements. They often find it difficult to feed themselves and in later stages have difficulty in eating even when they are fed by others. As a result, they often deteriorate in their nutritional status and develop failure to thrive. The resulting malnutrition leads to impaired immunity and increased susceptibility to infection, pressure ulcers and delayed wound healing. Eating or swallowing difficulties also lead to increased risk of pneumonia and higher mortality rates. According to literature, 13-86% of patients with advanced dementia have an eating or swallowing problem [2-4].

Feeding options in advanced dementia

In the late stages of dementia, patients lose their ability to feed on their own and are unable to meet their nutritional needs. The families of such patients are faced with discussing the two available options in continuing care: the first is to continue to hand feeding orally with assistance, while fully understanding that intake could be inadequate and aspiration is possible; or to use enteral tube feeding, which also carries similar risks. Two forms of enteral tube feeding are commonly used in patients with feeding difficulties: nasogastric (NG) tube which is passed through the nose into the stomach and percutaneous endoscopic gastrostomy (PEG) tube which is placed directly into the stomach through an incision in the abdominal wall with the help of an endoscope. The NG tubes are uncomfortable, not well tolerated, their insertion may be resisted, they are often dislodged, and hence seen as a less practical long-term option outside of hospitals. PEG tube feeding usually becomes perceived as the method of choice if the feeding plan is long term. The decision to use artificial nutrition in patients with dementia is controversial and complex due to the emotional aspects and ethical dilemmas that are often involved [5].

## Materials and methods

We prepared a questionnaire consisting of 15 questions related to physicians’ perspectives on PEG tube utilization and outcomes in patients with advanced dementia. The questionnaire was sent out in January 2019 to experienced physicians specialized in gastroenterology or general medicine, working in hospital or primary care settings in various countries including Australia, India, United Arab Emirates (UAE), United Kingdom (UK) and United States of America (USA). The data were collected in January and February of 2019 and the responses were analyzed. The physicians were also interviewed verbally to ensure their understanding of the purpose of data collection. The questionnaire used is shown in Table 1 below.

**Table 1:** Questionnaire PEG: Percutaneous endoscopic gastrostomy; QOL: Quality of life

International Survey of Physicians’ perspectives on PEG Tube in Dementia patients
1, Improves the QOL:
a. Strongly agree b. Agree c. Neither agree nor disagree d. Disagree e. Strongly disagree
2. Prolongs survival:
a. Strongly agree b. Agree c. Neither agree nor disagree d. Disagree e. Strongly disagree
3. Prevents pressure ulcers:
a. Strongly agree b. Agree c. Neither agree nor disagree d. Disagree e. Strongly disagree
4. Improves nutrition:
a. Strongly agree b. Agree c. Neither agree nor disagree d. Disagree e. Strongly disagree
5. Prevents aspiration:
a. Strongly agree b. Agree c. Neither agree nor disagree d. Disagree e. Strongly disagree
6. Have you seen mortality within 4 weeks of peg tube Insertion?
a. Yes b. No
7. How often do you hold a multidisciplinary meeting with patient’s family/surrogate decision maker prior to PEG tube placement?
a. Always b. Rarely c. Usually d. Sometimes e. Never
8. In your opinion who is driving the final decision on PEG tube placement?
a. Physician b. Surrogate or family c. Others (please specify)
9. Does insurance or Financial pressures influence decision on PEG tube placement?
a. Yes b. No
10. In your practice how often PEG tube is being placed when your recommendation was not to be placed?
a. Always b. Rarely c. Usually d. Sometimes e. Never
11. Do you consult and involve ethics team when there is a conflict in expectations and decisions regarding PEG tubes in dementia?
a. Always b. Rarely c. Usually d. Sometimes e. Never
12. Your Name: 13. Medical Profession:
14. In what country do you work:
15. I attest above information is provided by me and can be used for publication- pending peer review.
a. Yes b. No

## Results

**Figure 1:**
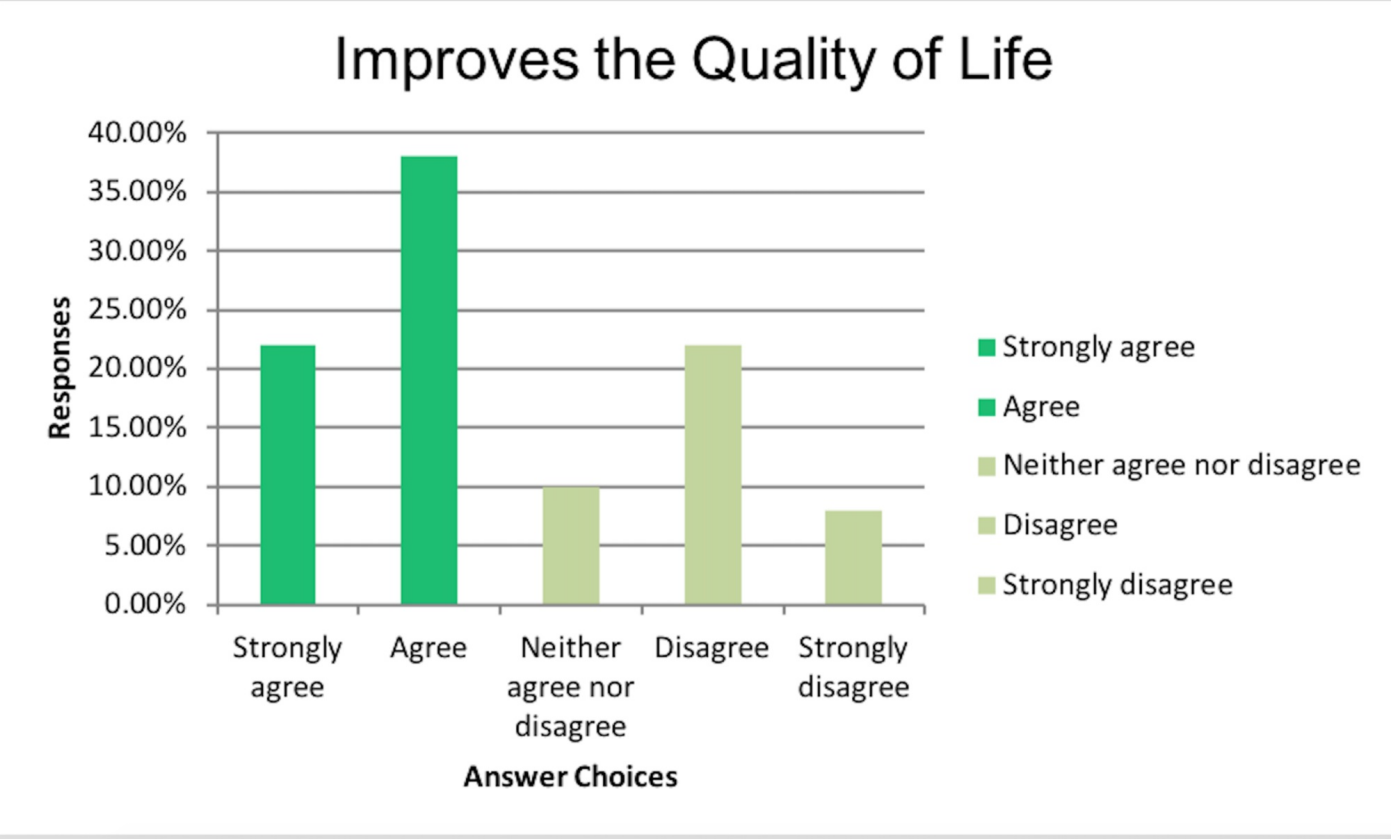
Improves the quality of life

We had 50 responses from physicians in various countries. The most responses came from India (42%, 21 of 50), Australia (22%, 10 of 50) and the UK (18%, 9 of 50). A total of 75.51% (37 of 50) were Gastroenterologists, and the rest (12.24%, 6 of 50) were Acute care physicians and General practitioners (12. 24%, 6 of 50). In our survey, surprisingly, 60% (30 of 50) of the participating physicians believed that PEG tubes improved the ‘Quality of Life’ (QOL) of patients with advanced dementia as shown in Figure 1 below.

**Figure 2:**
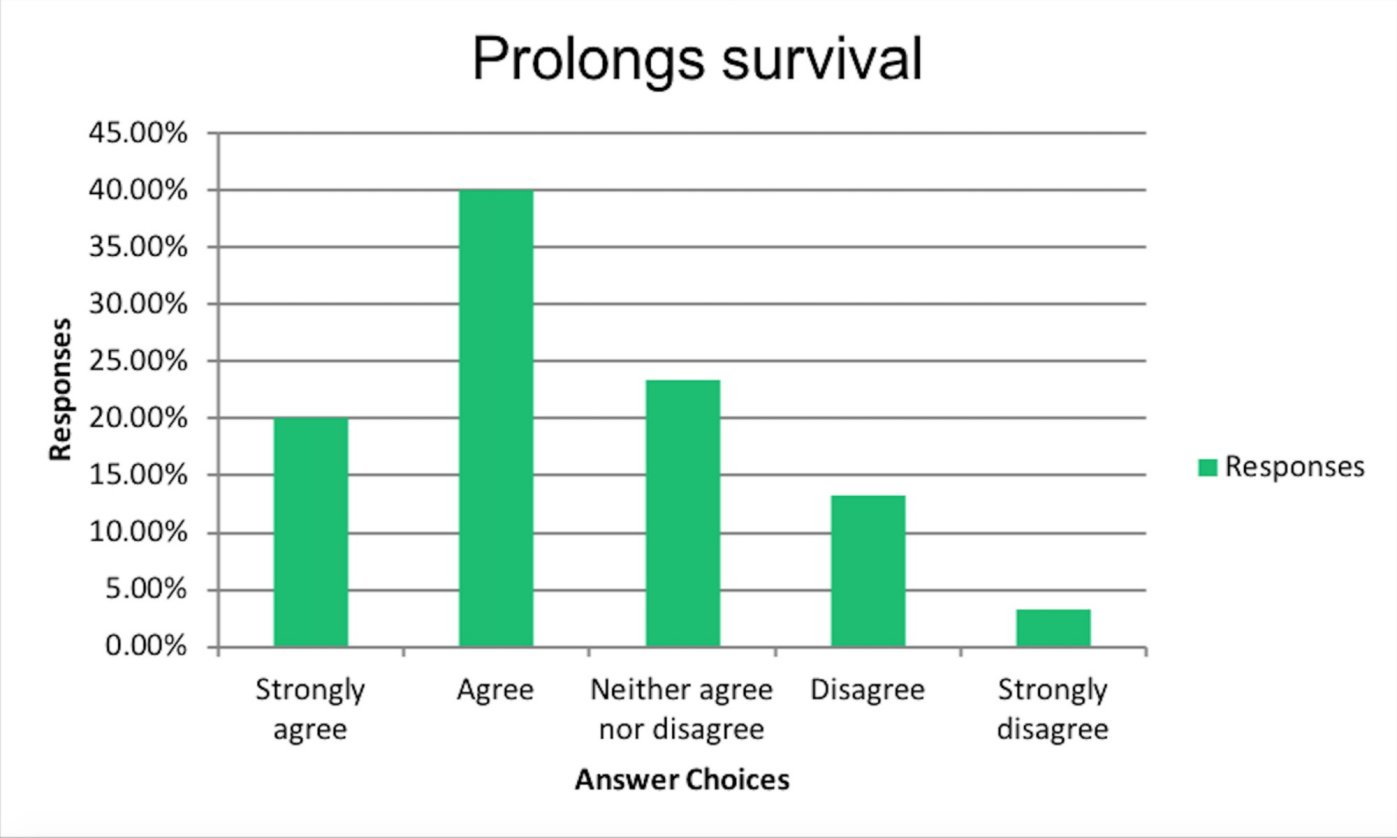
Prolongs survival

In order to further evaluate the reasons and have a better understanding of the subset of physicians who believed improved QOL with PEG tubes in dementia, we performed subset analysis. The results of the subset analysis are summarized as follows. Amongst physicians who believed in improved QOL, 60% believed PEG tubes also prolong survival as shown in Figure 2.

**Figure 3:**
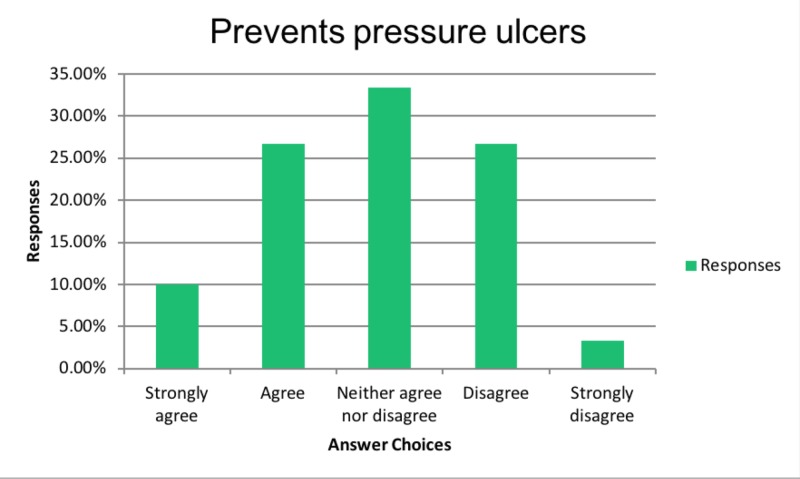
Prevents pressure ulcers

In the same subset of physicians who believed in improved QOL, 37% believed that PEG tubes also prevent pressure ulcers as shown in Figure 3.

**Figure 4:**
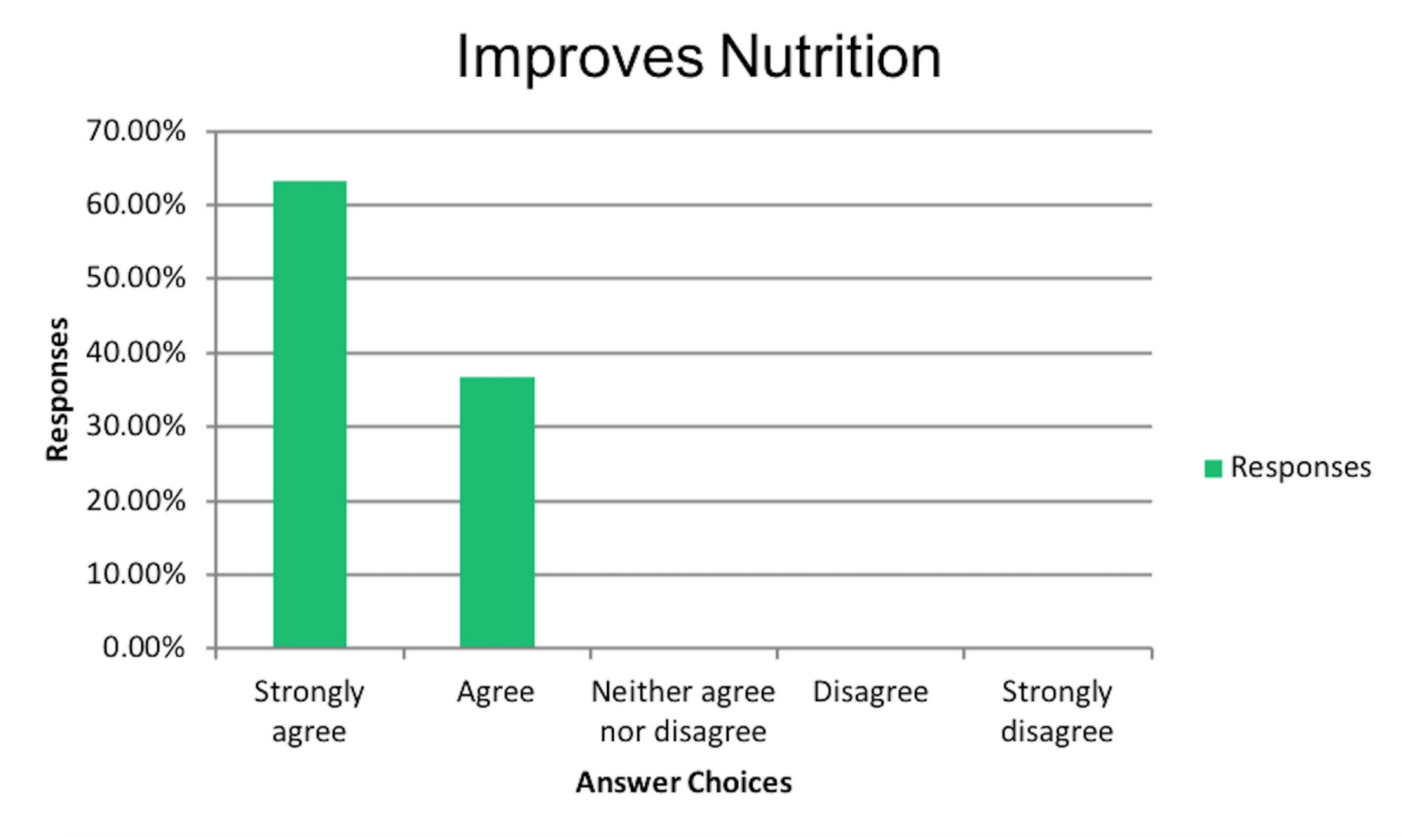
Improves nutrition

Similarly, physicians who believed in improved QOL with PEG tubes also believed in improved nutritional status as shown in Figure 4.

**Figure 5:**
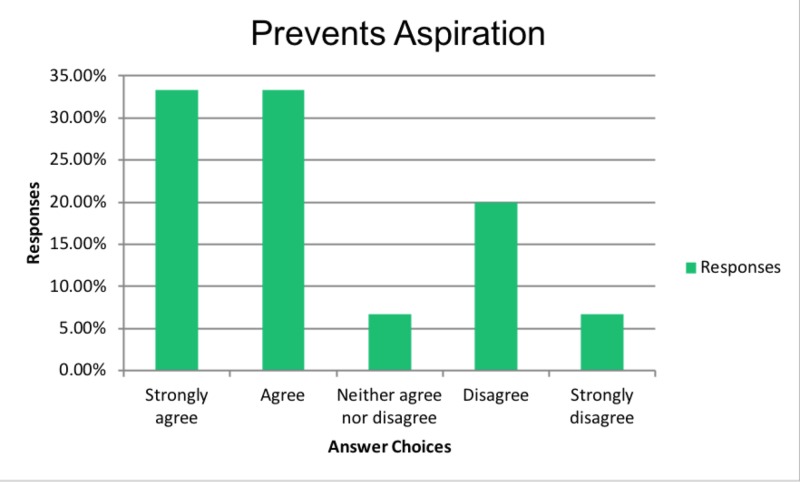
Prevents aspiration

Lastly, 67% of those who believed in improved QOL, also believed that PEG tubes prevent aspiration pneumonia as shown in Figure 5.

These misperceptions could have led the physicians to falsely believe the effect on overall QOL. Table 2 illustrates the frequency of responses for key variables.

**Table 2:** Frequency for key variables N: Total number; QOL: Quality of Life

	N	Strongly Agree	Agree	Neutral	Disagree	Strongly Disagree
Improves the QOL	50	11	19	5	11	4
Prolongs survival	50	7	19	10	9	5
Prevents pressure ulcers	50	3	12	13	18	4
Improves nutrition	50	20	24	4	1	1
Prevents aspiration	50	10	12	4	16	8

Table 3 provides descriptive statistics (the mean and standard deviation) for the key variables in our survey. 

**Table 3:** Descriptive statistics for key variables PEG: Percutaneous endoscopic gastrostomy; N: Total number; QOL: Quality of life

	N	Mean	Std. Deviation
Improves the QOL	50	2.56	1.28
Prolongs survival	50	2.72	1.21
Prevents pressure ulcers	50	3.16	1.08
Improves nutrition	50	1.78	0.84
Prevents aspiration	50	3	1.43
Have you seen mortality within 4 weeks of PEG tube insertion?	50	1.62	0.49
How often do you hold a multidisciplinary meeting with patient's family/surrogate decision maker prior to PEG tube placement?	50	2.06	1.11
In your opinion who is driving the final decision on PEG tube placement	48	1.5625	0.65
Does insurance or financial pressures influence decision on PEG tube placement	50	1.76	0.43
In your practice how often PEG tube is being placed when your recommendation was not to be placed	50	3.62	1.01
Do you consult and involve ethics team when there is a conflict in expectations and decisions regarding PEG tubes in dementia?	50	2.96	1.44

Table 4 presents the correlations between QOL and the other key variables of the study. The findings clearly suggest that most participants perceive that the PEG tube improves the QOL of the participants because they believe that the PEG tube prolongs survival (r=.54, p<.01), prevents pressure ulcers (r=.39, p<.01), improves nutrition (r=.65, p<.01), and prevents aspiration (r=.59, p<.01).

**Table 4:** Correlation table QOL: Quality of life

	1	2	3	4	5
1. Improves the QOL	1				
2. Prolongs survival	.537**	1			
3. Prevents pressure ulcers	.393**	.473**	1		
4. Improves nutrition	.648**	.499**	.446**	1	
5. Prevents aspiration	.591**	.436**	.598**	.527**	1

Similarly, when perceptions on mortality were analyzed in our survey, it was noted, that 52%, (26 of 50) agreed that PEG prolonged survival while 28% (14 of 50) did not agree and 20% (10 of 50) chose to remain neutral. In the subset of physicians who believed that the PEG prolonged survival, it was noted that all of them also believed that it improved the nutritional status and QOL.

Most physicians in this survey felt that PEG placement did not prevent pressure ulcers (44%, 22 of 50), and only 30% (15 of 50) thought it prevented this from happening and 26% (13 of 50) did not have a definite opinion on this issue.

An overwhelming majority 88% (44 of 50) of the physicians in our survey, felt PEG tube placement improved the nutrition of patients with dementia and only 4% (two of 50) disagreed and 8% (four of 50) remained neutral.

48% (24 of 50) thought PEG tube does not prevent aspiration, but 44% (22 of 50) thought it did, and 8% (4 of 50) were unsure.

Only 38% (16 of 50) had seen mortality in demented patients within 4 weeks of PEG placement whereas 62% (38 of 50) hadn’t.

70% (35 of 50) physicians felt that there was a need to hold multidisciplinary meetings with the patient’s family or surrogate member before PEG tube placement whereas 14% (7 of 50) hardly ever practiced this.

The majority (62 %, 31 of 50) felt physicians were driving the final decision on PEG placement, while 30% (15 of 50) felt surrogate or family members were in charge of this decision. 6% (three of 50) felt no particular person was in charge of the final decision and 2% (one of 50) thought that the final decision was to be jointly taken by the physician and family members of the patient.

76% (38 of 50) did not feel insured, or financial pressures influenced their decision on PEG tube placement whereas 24% (12 of 50) felt otherwise.

Fortunately 60% (30 of 50) physicians rarely or never had PEG tubes placed in patients with dementia against their recommendation but the rest 40% (20 of 50) had experience of this procedure being carried out in spite of their advice against it.

There was quite a mixed opinion on the involvement of ethics team in the decision regarding PEG placement in patients with dementia. 42% (21 of 50) usually or always made use of the ethics team while 18% (36 of 50) hardly or never used their help and 22% (11 of 50) used the ethics team on some occasions.

Physicians practicing in India mainly believed that placing a PEG tube in patients with dementia improved both their QOL and nutrition as seen by 100% of the response.

90% of physicians in Australia held Multidisciplinary Team (MDT) meetings before PEG placement, and 80% did not have a PEG tube placed against their recommendation.

## Discussion

In the American board of internal medicine (ABIM) led initiative of "Choosing Wisely" campaign, American Geriatric Society (AGS) has recommended against percutaneous feeding tubes in patients with advanced dementia and to encourage oral assisted feeding [6]. In severe dementia, there has been no difference in the outcomes such as death, aspiration pneumonia, functional status and patient comfort when patients are carefully hand-fed as compared to tube-fed patients. Tube feeding has been noted to be associated with agitation, increased use of physical and chemical restraints and worsening pressure ulcers. Despite these recommendations, tube feeding remains prevalent globally.

What is being practiced?

We can agree that the aim is always to improve the overall QOL, prevent aspiration pneumonia, reduce the risk of protein-calorie malnutrition and its potential consequences that include pressure ulcers, infection, starvation and death. But, is our practice evidence-based?

In a US survey, tube feeding was used for 34% of the 186,835 nursing home residents with advanced cognitive impairment [7]. However, according to Mitchell et al., the proportion of US nursing home residents with advanced dementia and eating dependency receiving feeding tubes decreased by approximately 50% between 2000 and 2014 [8]. In another cross-sectional survey of six long-term geriatric hospitals in Israel and Canada, the rate of tube feeding in patients with end‐stage dementia was 24% (92/2287), although there was a lower prevalence of feeding tube use in Canadian institutions than in Israeli hospitals [9]. In Israel, a study found knowledge gaps amongst physicians pertaining to feeding tube indications [10]. In Japan, the provision of artificial nutrition and hydration has been long considered standard care even in individuals with severe cognitive impairment due to multiple reasons including, and not limited to the national health insurance system that allows elderly patients to become long-term hospital in-patients, legal and emotional barriers with regard to limiting treatment options, the risk of prosecution, cultural barriers, all promoting PEG placement [11]. 

Is percutaneous endoscopic gastrostomy the right choice in the context of outcomes?

Placement of PEG tube in older adults was shown to have significant mortality and morbidity [12]. Despite this, physicians seem to have accepted PEG tube placement as a relatively benign procedure. Minor procedure-related complications including pain, abdominal wall ulcers, wound infections, peristomal leakage, and tube displacement may occur in approximately 14% of cases. Some of the more severe procedure-related complications including hemorrhage, bowel or liver perforation and aspiration are also possible and reported to have occurred in 3% of cases [13]. Despite the widespread use of PEG tube for feeding in patients with dementia, the bulk of the evidence does not demonstrate long-term benefits in reducing aspiration risk, healing of pressure ulcers, prolonging survival, or improving QOL [14].

Risk of aspiration pneumonia

Studies have failed to show prevention or reduction aspiration pneumonia with PEG tubes. Colonization of the oropharynx with pathogenic bacteria was seen more often in NG or PEG tube fed elderly patients when compared to the orally fed patients [15]. Therefore, the use of PEG tubes might put them at higher risk for pathogenic inoculation which is thought to be the origin of true aspiration pneumonia. A retrospective review of 79 patients who had PEG or percutaneous endoscopic jejunostomy (PEJ) showed that aspiration occurred in 11.4% and that aspiration risk was not reduced further in those with PEJ [16]. PEG tube placement has also shown to decrease lower esophageal sphincter tone, potentially increasing regurgitation risk [17]. Several strategies such as proper positioning, modified diets, oral hygiene, or delivery of food have been proposed as a measure to prevent aspiration pneumonia; but, through a systematic review, it was noted that the data is insufficient to determine the effectiveness of these proposed strategies [18]. Small randomized trials have shown no decrease in aspiration risk with post-pyloric versus gastric feeding tubes or NG versus percutaneous feeding tubes. This risk of aspiration is no different in continuous or intermittent tube feeds. Because of the lack of evidence to support aspiration prevention, it has been recommended to periodically address goals of care before initiating tube feeding [19].

Risk of pressure ulcers

There are no studies which show that providing nutrition through feeding tubes prevent pressure ulcer formation. A 2-year study in nursing home residents with dementia showed no significant survival benefit from nutrition with a feeding tube as compared to those without one [20]. Presence of a pressure ulcer was noted to be one of the independent risk factors associated with feeding tube placement in the same study. Another study by Teno et al. showed that hospitalized nursing home residents receiving a PEG tube were 2.27 times more likely to develop a new pressure ulcer, and those with a pressure ulcer were less likely to have the ulcer heal when they had a PEG tube inserted [21].

Nutritional status

There was no improvement in functional or nutritional status after PEG placement in patients with dementia according to one retrospective study by Kaw et al. [22]. Another study which looked at the long-term outcome after PEG in community setting showed that over 70% of the 150 patients had no improvement in nutritional, functional or subjective health status [23].

Survival and mortality

PEG tube feeding did not have any impact on decreasing mortality in patients with dementia. Co-morbidities often influence survival in patients following PEG placement more than dementia. A retrospective study at a tertiary academic center of 392 patients who underwent PEG tube placement, including 165 patients with dementia showed that neither short-term nor long-term mortality improved, these patients had a shorter time to death and had no improvement in albumin [24]. In a retrospective cohort survival analysis of 361 consecutive patients requiring PEG feeding in two District General Hospitals in the UK between 1992 and 1997, it was found that there is a high initial mortality of 28% at 30 days and in patients with dementia the 30-day mortality was 54% and 1-year mortality was 90% [25]. 

Decision to place a percutaneous endoscopic gastrostomy tube

Oral route of feeding remains the most satisfying and universally recommended feeding route in patients with advanced dementia and PEGs are often placed prematurely before exploring every alternate option for oral feeding. One of the initial steps should be to ascertain the reason(s) for inability to swallow through a multidisciplinary team approach, including involvement of neurologist, occupation therapist (and speech and swallow specialist), and gastroenterologist [26]. Diets should be modified for proper consistency and tailored to patient’s preferences and QOL, with a provision of help from staff or caregiver. Advance Directives, when available, could provide information about their expectations and details regarding preferred feeding options, especially concerning artificial nutrition. In case this information is unavailable, decisions regarding PEG tube should follow meaningful and timely discussions between patient or caregiver and a multidisciplinary team who should discuss both short-term and long-term outcomes without providing undue expectations [27]. In cases where there are no advance directives or surrogates and where there is a conflict of opinion between treating physician and patient’s surrogate or family, consultation with the Ethics committee would be prudent before placing PEG tube.

Recommendations versus physicians’ perspectives

Most professional organizations across the world do not recommend the use of feeding tubes in patients with advanced dementia [6, 28-29]. Despite this, physicians seem to have varied opinions and practice regarding the feeding tubes in patients with dementia and its effects on these patients [10-11]. Our study looked at the perspectives of fully licensed and experienced gastroenterologists, acute care, and general practitioners about multiple aspects of PEG tubes in patients with dementia. To our knowledge, there are no international surveys exploring physicians perspectives about PEG tubes in dementia globally. The surveyed physicians in our study were from various countries who cared for patients having dementia with feeding difficulties.

## Conclusions

Our survey was done to investigate further the contributing factors for this high global prevalence with a focus on the practitioner’s perceptions regarding PEG tubes in dementia. The misperception seems to be a significant global problem as noted in our survey. Professional societies both international and regional, in various countries should take initiatives similar to Choose Wisely campaign led by ABIM in conjunction with AGS to educate and guide physician leaders and communities. This crisis also calls for more innovative solutions to ensure family engagement and physician alignment for individualized high quality care and avoiding procedures that may not be necessary. Continued medical education for practicing clinicians in this aspect of elderly care and raising awareness in the community regarding Advance Directives may be some of the key strategies. Multidiciplinary team meetings and clinical ethics consultation would be useful in situations where there are conflicting opinions
